# RAG_MCNNIL6: A Retrieval-Augmented Multi-Window Convolutional Network for Accurate Prediction of IL-6 Inducing Epitopes

**DOI:** 10.1021/acs.jcim.4c02144

**Published:** 2025-02-19

**Authors:** Cheng-Che Chuang, Yu-Chen Liu, Wei-En Jhang, Sin-Siang Wei, Yu-Yen Ou

**Affiliations:** † Department of Computer Science and Engineering, Yuan Ze University, Chung-Li 32003, Taiwan; ‡ Graduate Program in Biomedical Informatics, Yuan Ze University, Chung-Li 32003, Taiwan

## Abstract

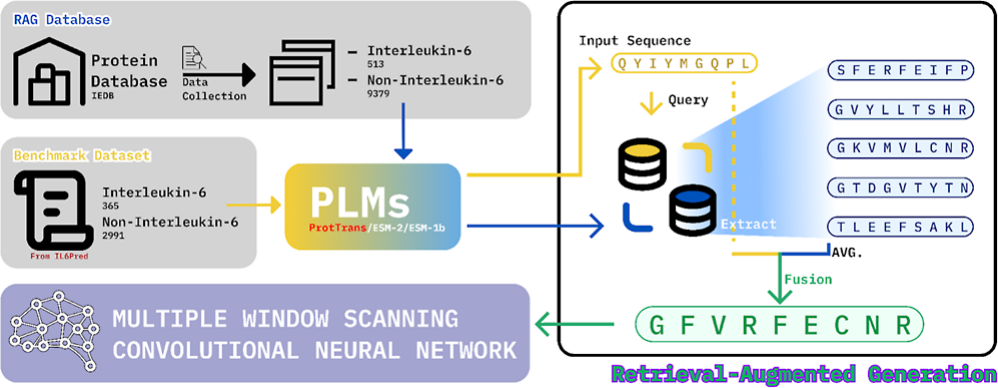

Interleukin-6 (IL-6) is a critical cytokine involved in immune regulation, inflammation, and the pathogenesis of various diseases, including autoimmune disorders, cancer, and the cytokine storm associated with severe COVID-19. Identifying IL-6 inducing epitopes, the short peptide fragments that trigger IL-6 production, is crucial for developing epitope-based vaccines and immunotherapies. However, traditional methods for epitope prediction often lack accuracy and efficiency. This study presents RAG_MCNNIL6, a novel deep learning framework that integrates Retrieval-augmented generation (RAG) with multiwindow convolutional neural networks (MCNNs) for accurate and rapid prediction of IL-6 inducing epitopes. RAG_MCNNIL6 leverages ProtTrans, a state-of-the-art pretrained protein language model, to generate rich embedding representations of peptide sequences. By incorporating a RAG-based similarity retrieval and embedding augmentation strategy, RAG_MCNNIL6 effectively captures both local and global sequence patterns relevant for IL-6 induction, significantly improving prediction performance compared to existing methods. We demonstrate the superior performance of RAG_MCNNIL6 on benchmark data sets, highlighting its potential for advancing research and therapeutic development for IL-6-mediated diseases.

## Introduction

Interleukin-6 (IL-6) stands as a pivotal cytokine orchestrating diverse physiological processes, ranging from immune responses and inflammation to hematopoiesis.^1−3^ While crucial for maintaining homeostasis, aberrant IL-6 expression is implicated in a wide spectrum of diseases, including autoimmune disorders, chronic inflammatory conditions, cancer, and the life-threatening cytokine storm observed in severe infections like COVID-19.^4−7^ Understanding the mechanisms underlying IL-6 induction and developing targeted therapeutic interventions are essential for addressing these health challenges.

A key aspect of IL-6’s biological activity lies in its interaction with specific antigenic epitopes, the short peptide fragments derived from pathogens or self-antigens that trigger the immune response and subsequent IL-6 production.^8^ Identifying these IL-6 inducing epitopes is paramount for understanding the molecular basis of IL-6 driven pathologies and for developing epitope-based vaccines and immunotherapies.^9,10^ Therefore, accurately identifying these IL-6 inducing epitopes is not only crucial for understanding disease mechanisms but also holds significant promise for the rational design of effective epitope-based vaccines and immunotherapies. However, while IL-6 plays a critical role in immune responses, it is also implicated in the pathogenesis of various diseases, including the cytokine storm associated with severe infections.^11^ Therefore, precise identification of IL-6 epitopes, with an understanding of their induction levels, is crucial for the safe and effective design of epitope-based vaccines and immunotherapies.

Recent years have witnessed significant advances in computational approaches for epitope prediction, driven by the rise of machine learning and deep learning techniques. Models like MVIL6^12^ and StackIL6^13^ have demonstrated improvements in prediction accuracy compared to earlier methods. MVIL6 utilizes a MG-BERT^14^ approach to capture different aspects of epitope sequences, while StackIL6 uses a stacking approach to generate new features. However, despite their success, these models often struggle to fully capture the complexity of protein-epitope interactions. Specifically, they mainly rely on single sequence-based features and may fail to incorporate crucial contextual information necessary for accurate epitope recognition. This challenge is particularly evident when dealing with complex protein-epitope interactions that involve both local sequence motifs and long-range dependencies.

The recent advent of deep learning, particularly pretrained protein language models (PLMs) like ProtTrans,^15^ has revolutionized the field of bioinformatics, enabling the extraction of rich embedding representations that capture intricate patterns and relationships within protein sequences.^15−17^ While PLMs excel at capturing global sequence features, they may not fully exploit the wealth of information present in homologous sequences and functional contexts, potentially limiting their ability to discern subtle local patterns crucial for epitope recognition.

To address these limitations, we propose RAG_MCNNIL6, a novel deep learning framework that integrates Retrieval-augmented generation (RAG)^18−20^ with multiwindow convolutional neural networks (MCNNs)^21−25^ for accurate and rapid prediction of IL-6 inducing epitopes. RAG_MCNNIL6 leverages PLM embeddings and incorporates a RAG-based similarity retrieval strategy to enrich peptide sequence representations with relevant contextual information from a curated database of protein sequences. The MCNN architecture further enables the model to effectively capture both local and global sequence patterns critical for IL-6 induction. We hypothesize that this innovative approach will outperform existing epitope prediction methods and provide a valuable tool for advancing research and therapeutic development for IL-6-mediated diseases.

## Materials and Methods

### Data Collection

As part of this study, experimentally verified interleukin-inducing peptides were extracted from the Immune Epitope Database (IEDB)^26^ to build a RAG database relevant to IL-6-inducing peptides. This data set includes 513 peptides labeled as IL-6-inducing (positive). Additionally, 9379 peptides labeled as non-IL-6-inducing or inducing other interleukins (negative) were incorporated into the RAG database to create a more comprehensive and diverse data set.

To conduct training and testing, we have selected the benchmark data set compiled by the developers of IL6Pred.^27^ In order to ensure a consistent basis for comparison, both the positive and negative data sets were derived from the original study. As shown in Table 1, the benchmark data set was divided into two sets, a training set consisting of 292 IL-6-inducing peptides and 2393 non-IL-6-inducing peptides, and a validation set consisting of 73 IL-6-inducing peptides and 598 non-IL-6-inducing peptides.

**Table 1:** Statistics of the Survey Dataset

data set	total	IL-6-inducing peptides	non-IL-6-inducing peptides
training data	2685	292	2393
validation data	671	73	598
RAG^a^ database	9892	513	9379

a RAG(Retrieval-augmented generation).

### Retrieval-Augmented Generation (RAG) Framework for Protein Sequence Representation

**Figure 1:**
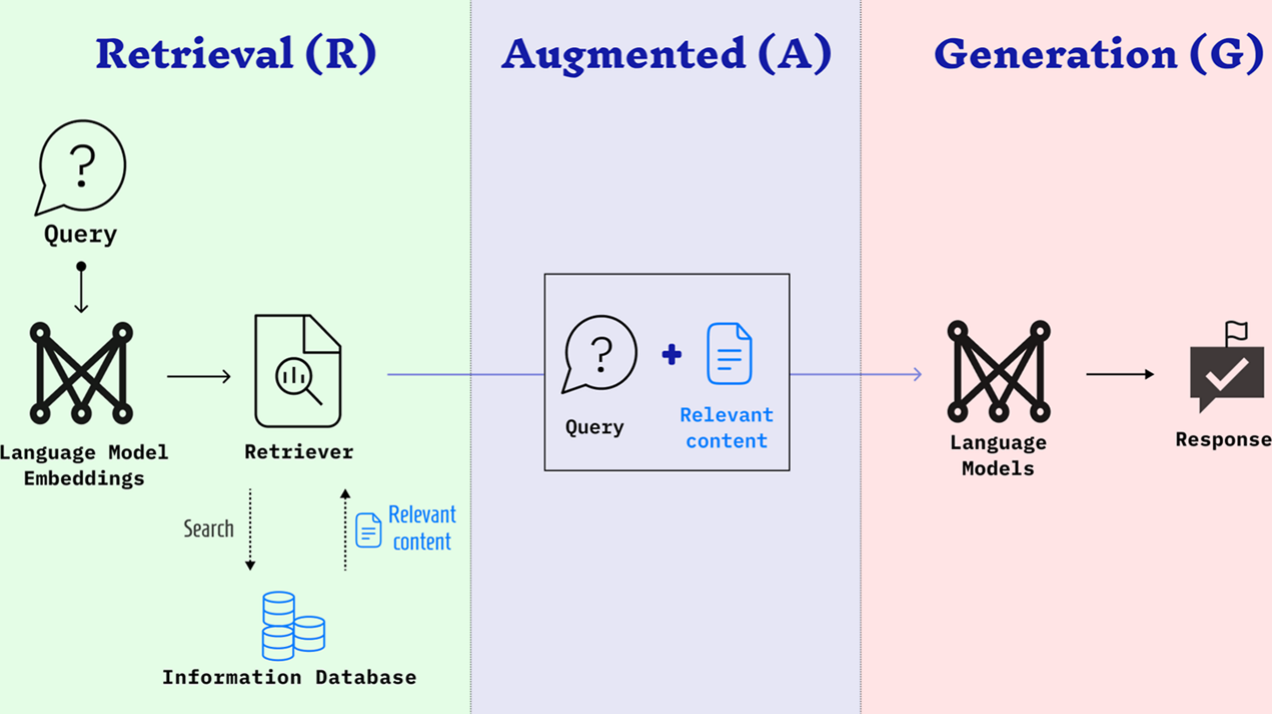
General framework for Retrieval-augmented generation.

In recent years, large language models (LLMs) have made remarkable strides in natural language processing (NLP) tasks,^28,29^ largely due to their ability to extract deep contextual understanding from extensive data sets. A key innovation driving this progress is the Retrieval-augmented generation (RAG) framework, which enhances LLMs by integrating relevant information from external databases into the generated output. As Figure 1 shows, The RAG framework starts with an input query, which is transformed into an embedding and compared against a prebuilt vector store containing relevant sequences or data. The system retrieves the most similar sequences from this database, which provide valuable contextual information. These retrieved sequences are then fused with the original query to form a richer prompt. This enhanced prompt is fed into a LLM that generates an output based on both the original query and the retrieved context. By integrating both stored knowledge and real-time contextual retrieval, RAG significantly improves the accuracy and reliability of many tasks.

We applied the RAG framework to protein sequence analysis, enabling the retrieval of relevant protein sequences and the incorporation of functional contexts from a curated database. For this study, we selected peptides that induce IL-6 as well as other interleukin-induced peptides. In the next step, each sequence was then transformed into high-dimensional embeddings using PLM, a protein language model based on self-supervised pretraining on raw protein sequences.

**Figure 2:**
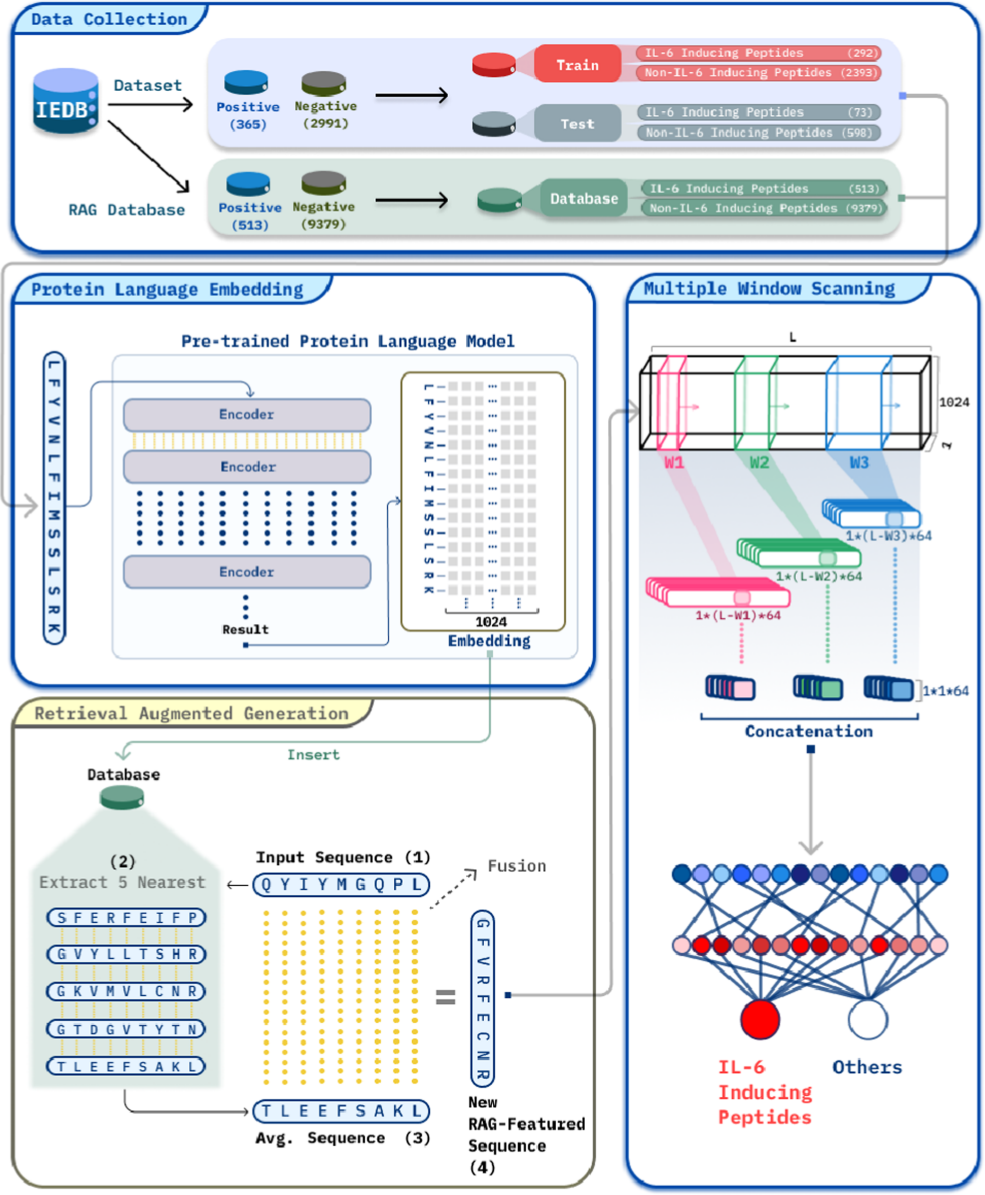
A flow diagram of the Retrieval-augmented generation framework for the RAG_MCNNIL6 model.

Illustrated in Figure 2, by feeding protein sequences into PLMs, input sequences would be transformed into high-dimensional embedding vectors loaded with extensive global and local contextual information. With this approach, using ProtTrans,^15^ we obtain *L* × 1 × 1024 matrices, where “*L*” denotes the sequence length, which allows us to extract both the linguistic patterns of a protein and its biochemical properties.

**Figure 3:**
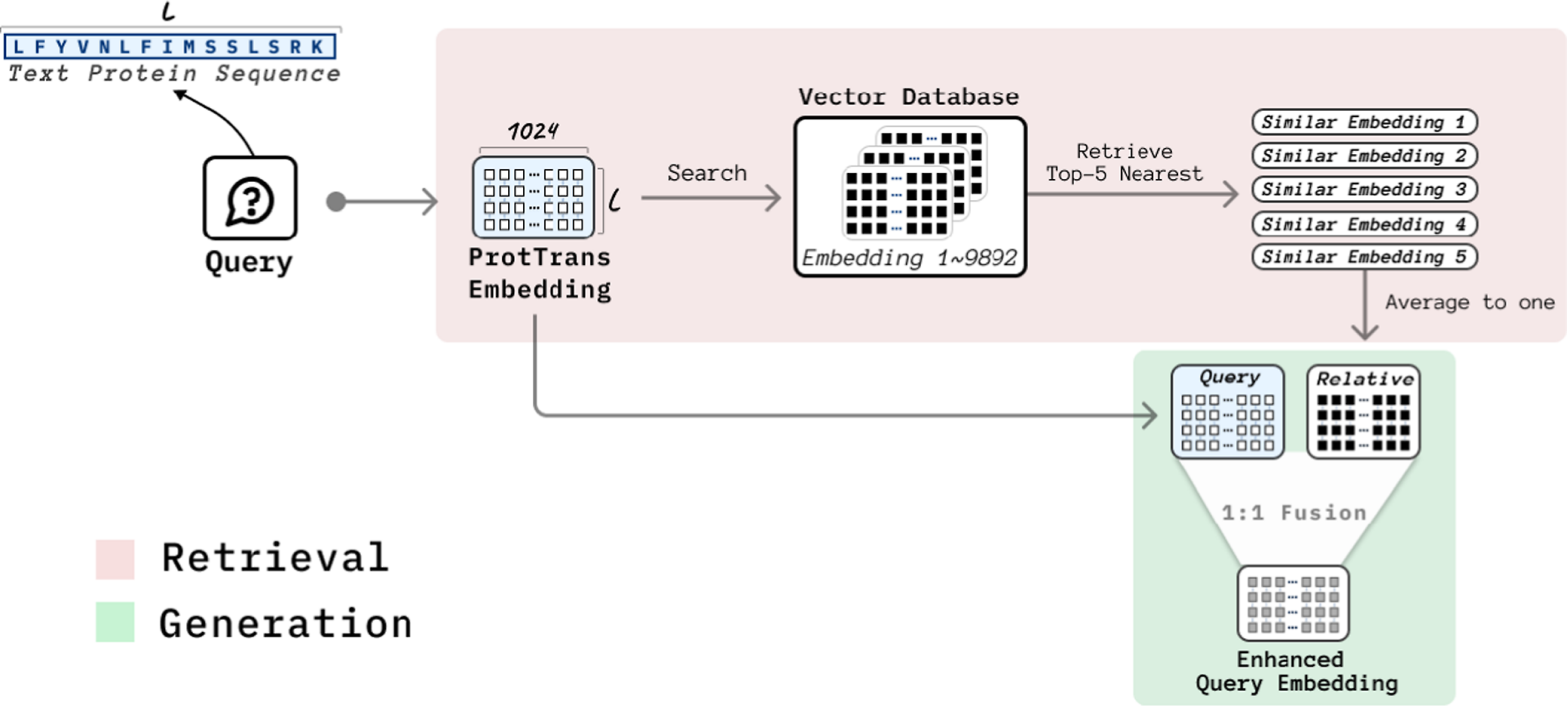
Retrieval-augmented generation framework for protein sequences.

Once the embeddings were computed, they were compared against those stored in our database using a distance metric to measure similarity. As shown in Figure 3. By retrieving and averaging the embeddings of the top five most similar sequences, we obtained additional contextual information that enriched our understanding of the query sequence. The fusion process combined the original query sequence embedding with the averaged embeddings from similar sequences in a 1:1 weighting ratio. This approach ensures that both the unique features of the original sequence and the contextual information from similar sequences are equally valued.

This enhanced representation significantly improves the model’s ability to recognize subtle differences in sequence function, thus advancing the accuracy and reliability of complex tasks like epitope identification. The RAG framework effectively combines retrieval and generation processes, beginning with the transformation of an input query into an embedding. This results in a more robust and comprehensive sequence representation, which can capture a wider range of information than the original embedding alone could.

### Multiple Window Scanning Deep Learning Networks Architecture

The multiwindow scanning technique was originally developed for CNN-based sentence classification tasks.^22^ In 2018, the Deepfam^23^ team adapted it for protein sequence analysis. By 2022, the MCNN-ETC^24^ model successfully applied multiwindow scanning along with attribute sets generated from multiple sequence alignments. Building on this progress, in 2023, we began utilizing multiwindow scanning technology in combination with pretrained protein language models (PLMs) to further advance protein sequence analysis.^25^

In this study, we present an innovative deep learning approach that integrates RAG with multiwindow scanning. Our method uses multiple convolutional layers with varying window sizes in a multiwindow scanning architecture to process enhanced embeddings from the RAG framework. For example, a 16 × 1 × 1024 convolution kernel is applied to an *L* × 1 × 1024 input matrix, where *L* represents the sequence length and 1024 corresponds to the number of embedding channels at each position. This input, a 3D matrix, captures the enhanced embeddings (*L* × 1 × 1024) for every amino acid in the sequence. By scanning this matrix with 1D convolution kernels of different window sizes (e.g., 16 residues), each convolutional layer focuses on specific sequence patterns. In this case, the 16 × 1 × 1024 kernel captures 16 adjacent amino acids. After the convolution operation, the 3D input is reduced to a 2D matrix of size *L* × 1, where *L* remains the sequence length and 1 represents the number of filters used. This step extracts the most significant features from each scanning window. A max pooling layer then reduces the *L* × 1 output of each filter to a 1 × 1 vector, retaining only the most dominant features.

By employing convolutional layers with different window sizes, the model is able to analyze both local and global patterns in the sequence. The flattened outputs from each convolution window are concatenated to form a comprehensive feature vector, representing the cross-scale patterns in the sequence. This multiscale analysis of enhanced embeddings from the RAG framework enables the multiwindow scanning architecture to effectively capture essential properties of protein sequences for classification tasks.

### Performance Evaluation

The performance of the model was evaluated using various established metrics. These evaluation metrics included sensitivity, specificity, accuracy, and the Matthews correlation coefficient (MCC). We also selected the commonly used evaluation indicators composed of balanced accuracy (BACC) in order to conduct a comparative study with the previous method. These metrics were derived using Formulas 1–5, described below
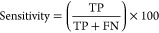
1
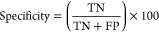
2
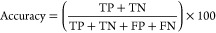
3
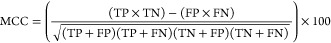
4
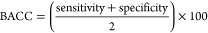
5

In this context, FP represents false positive, FN represents false negative, TP represents true positive, and TN represents true negative.

In addition, we also evaluated the performance of the model using the area under the receiver operating characteristic curve (ROC-AUC), a commonly used metric for evaluating classification models. The AUC measures the ability of the model to distinguish between classes, with a value ranging from 0 to 1. A higher AUC indicates better precision and discriminative power for model classification, whereas a lower value reflects subpar performance.

## Results

This section evaluates the performance of the RAG feature set combined with the multiwindow CNN model for classifying IL-6-inducing peptides, utilizing indicators such as sensitivity, specificity, accuracy, Matthews correlation coefficient (MCC), and Area under the ROC curve (AUC). A 5-fold cross-validation was implemented on the benchmark data set to fine-tune the model parameters, starting with single-window scans, which were later combined into a multiwindow configuration. The impact of varying the number of filters was analyzed to optimize the model. Furthermore, we compared the attribute sets generated by the RAG framework with different protein language models to highlight distinctions in feature representation. The robustness of the model was also tested against common classifiers. Finally, the model’s performance was benchmarked against previous studies, demonstrating improvements and advancements in IL-6 peptide classification.

### Comparison of Performance with Different Sizes of Single Windows

To determine the optimal choice for multiple window combinations, we first evaluated the effect of using different sizes of single windows ranging from 2 to 34. As shown in Table 2, the smallest window size of 2 demonstrates the highest MCC of 0.5436 and an AUC of 0.9023, reflecting strong initial discriminative ability. While AUC initially declines as the window size increases, it peaks at 34 with the highest AUC of 0.9072, indicating optimal discriminative power at this size. Sensitivity is highest at window size 2 (0.8842) and peaks again at 24 (0.8590), while specificity remains relatively stable but slightly improves at larger sizes such as 34 (0.8323). Balanced accuracy (BACC) follows a similar trend, with high values at sizes 2 and 34. Although smaller windows excel in MCC and short-sequence pattern recognition, larger sizes like 34 deliver more robust overall performance, especially in AUC and accuracy, highlighting a trade-off between smaller windows’ precision and larger windows’ discriminative capability.

**Table 2:** Comparison of Performance with Different Sizes of Single Windows

window	BACC	sensitivity	specificity	accuracy	MCC	AUC
2	0.8644	0.8842	0.8446	0.8482	0.5436	0.9023
4	0.8467	0.8475	0.8458	0.8464	0.5204	0.8764
8	0.8112	0.8144	0.8079	0.8086	0.4448	0.8362
16	0.8320	0.8342	0.8298	0.8302	0.4838	0.8781
24	0.8368	0.8590	0.8146	0.8197	0.4834	0.8792
32	0.8351	0.8476	0.8226	0.8255	0.4855	0.8898
34	0.8516	0.8709	0.8323	0.8369	0.5179	0.9072

### Comparison of Performance with Different Combinations of Windows

After evaluating the performance of a single window, we evaluated the performance of multiple window combinations in order to determine whether combining different window sizes would enhance the model’s overall performance. Starting with a single window size of 34, we then add windows based on the performance of the AUC from Table 2. The results indicate that combining multiple window sizes improves the model’s overall performance, with the most significant improvements observed when window sizes 2, 34, 32, 24, 16, and 4 are used together. As shown in Table 3, the combination of 2, 4, 16, 24, 32, 34 achieves the highest MCC of 0.5618 and AUC of 0.9132, along with the highest BACC of 0.8760 and strong sensitivity (0.8998) and specificity (0.8521), indicating balanced and effective performance. While the addition of all tested windows (2, 4, 8, 16, 24, 32, 34) further improves sensitivity to 0.9029, the slight drop in specificity to 0.8258 suggests diminishing returns. Overall, the combination of six windows provides the most robust and consistent performance across key metrics.

**Table 3:** Comparison of Performance with Different Combinations of Windows

multiple window combination	BACC	sensitivity	specificity	accuracy	MCC	AUC
[34]	0.8516	0.8709	0.8323	0.8369	0.5179	0.9072
[34, 2]	0.8601	0.8866	0.8336	0.8391	0.5256	0.9037
[34, 2, 32]	0.8675	0.8848	0.8502	0.8536	0.5505	0.9028
[34, 2, 32, 24]	0.8689	0.8705	0.8672	0.8674	0.5611	0.9077
[34, 2, 32, 24, 16]	0.8503	0.8733	0.8272	0.8313	0.5139	0.8886
[34, 2, 32, 24, 16, 4]	**0.8760**	**0.8998**	**0.8521**	**0.8574**	**0.5618**	**0.9132**
[34, 2, 32, 24, 16, 4, 8]	0.8644	0.9029	0.8258	0.8343	0.5258	0.9063

### Comparison of Performance with Different Filter Numbers

The impact of different filter numbers on the model’s performance was then evaluated. Based on the number of filters adjusted between 128 and 1024, Table 4 shows how the model’s performance changes. It appears that using 1024 filters may result in overfitting, capturing excessive data and sacrificing specificity and overall performance balance. While 1024 filters achieve a high Sensitivity of 0.8982, they result in a lower Specificity (0.8233) and MCC (0.5196), indicating reduced performance balance. Conversely, a filter number of 512 provides the best overall performance across all metrics, achieving an MCC of 0.5618, an AUC of 0.9132, and strong BACC (0.8760), sensitivity (0.8998), and specificity (0.8521), demonstrating balanced and effective predictive power. Although 256 filters yield competitive specificity (0.8646) and MCC (0.5512), their overall performance is slightly lower than 512 filters. Thus, we have determined that 512 is the optimal filter number for maximizing predictive accuracy and discriminative power.

**Table 4:** Comparison of Performance with Different Filter Numbers

filters	BACC	sensitivity	specificity	accuracy	MCC	AUC
128	0.8586	0.8794	0.8379	0.8423	0.5269	0.9052
256	0.8608	0.8570	0.8646	0.8639	0.5512	0.9013
**512**	**0.8760**	**0.8998**	**0.8521**	**0.8574**	**0.5618**	**0.9132**
1024	0.8608	0.8982	0.8233	0.8311	0.5196	0.9043

### Comparison of Performance with Different Feature Sets

To assess the impact of the RAG-based similarity retrieval strategy, we also evaluated the multiwindow CNN model with ProtTrans embeddings, comparing its performance both with and without the integration of the RAG-based retrieval approach. In addition, we compared the feature sets generated by the RAG framework with those generated by other different protein language models to highlight the differences in feature representation. The methods included ESM-1b and ESM2 transformer protein language models developed by the meta fundamental AI research protein team (FAIR).

The results in Table 5 illustrate the baseline performance of ProtTrans, ESM-1b, and ESM-2 embeddings without the RAG framework in a 5-fold cross-validation setting. ProtTrans outperformed the other two models across all metrics, achieving the highest AUC (0.9132), BACC (0.8760), and MCC (0.5618), highlighting its superior ability to capture sequence-level and structural information. In comparison, ESM-2 and ESM-1b achieved lower AUCs of 0.8429 and 0.8372, respectively, alongside weaker MCCs and BACC, indicating less effective feature extraction for this task.

**Table 5:** Comparison of Performance with Different Feature Sets with 5-Folds Cross-Validation

feature sets	BACC	sensitivity	specificity	ACC	MCC	AUC
Cross-Validation (5-folds)						
**ProtTrans**	**0.8760**	**0.8998**	**0.8521**	**0.8574**	**0.5618**	**0.9132**
ESM-2	0.7806	0.7814	0.7797	0.7795	0.3999	0.8429
ESM-1b	0.7741	0.7837	0.7645	0.7676	0.3872	0.8372

**Figure 4:**
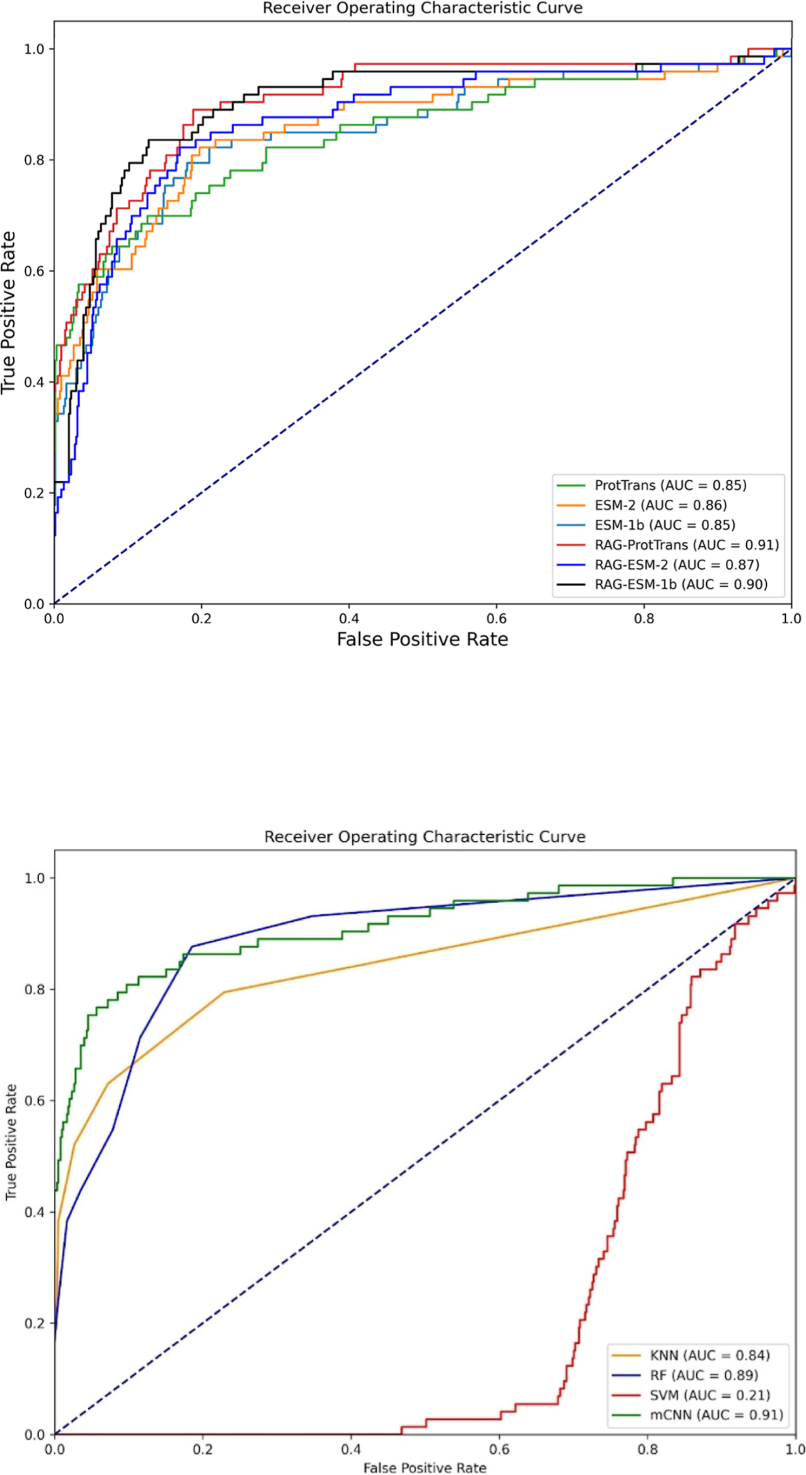
(a) ROC curve for the performance of predicting IL-6-inducing peptides with different feature sets. (b) ROC curve for the performance of predicting IL-6-inducing peptides with different classifiers.

As shown in Table 6, the results highlight the impact of integrating the RAG framework with different embeddings on an independent test data set. RAG-ProtTrans achieved the highest performance, with an AUC of 0.9074 and MCC of 0.6407, reflecting significant improvements over its baseline. While RAG-ESM-2 also demonstrated strong performance (AUC: 0.8995, MCC: 0.5446), its improvements were less pronounced than ProtTrans. RAG-ESM-1b exhibited moderate gains (AUC: 0.8680, MCC: 0.4739), suggesting potential for optimization. Notably, RAG-ProtTrans achieved the highest Specificity (0.9365) and ACC (0.9195), underscoring its robust discriminative power and compatibility with the RAG framework, further solidifying its position as the most suitable feature set for this study. A ROC curve for the performance of predicting IL-6-inducing peptides with different feature sets is presented in Figure 4a.

**Table 6:** Comparison of Performance with Different Feature Sets with Independent Test Dataset^a^

feature sets	BACC	sensitivity	specificity	ACC	MCC	AUC
Independent						
ProtTrans	0.7920	0.7260	0.8579	0.8435	0.4498	0.8502
ESM-2	0.7989	0.8219	0.7759	0.7809	0.4106	0.8599
ESM-1b	0.7954	0.8082	0.7826	0.7854	0.4089	0.8501
RAG-ProTrans	**0.8587**	**0.7808**	**0.9365**	**0.9195**	**0.6407**	**0.9074**
RAG-ESM-2	0.8534	0.8356	0.8712	0.8674	0.5446	0.8995
RAG-ESM-1b	0.8257	0.8219	0.8294	0.8286	0.4739	0.8680

a RAG(Retrieval-augmented generation).

### Comparison of Performance with Different RAG Combined Ratios

Table 7 compares the performance of the RAG framework with varying combine ratios of the original sequence embeddings to the RAG-retrieved sequence embeddings. The 1:1 ratio, where the original and retrieved embeddings contribute equally, achieved the highest overall performance, with an AUC of 0.9074, MCC of 0.6407, and the highest specificity (0.9365), indicating that equal weighting effectively balances the contributions of the two sources. This balance not only maximizes discriminative power but also delivers the highest accuracy (0.9195) and strong balanced accuracy (BACC) of 0.8587. These results underscore the effectiveness of an equal combination in leveraging complementary information from both original and retrieved embeddings.

**Table 7:** Comparison of Performance with Different RAG Combined Ratio

classifier	BACC	sensitivity	specificity	accuracy	MCC	AUC
**1:1**	**0.8587**	**0.7808**	**0.9365**	**0.9195**	**0.6407**	**0.9074**
1:2	0.8441	0.8219	0.8662	0.8614	0.5273	0.8943
1:5	0.8705	0.8630	0.8779	0.8763	0.5739	0.8931
2:1	0.8212	0.7945	0.8779	0.8689	0.5282	0.8638
5:1	0.8075	0.7671	0.8478	0.8390	0.4629	0.8596

In contrast, increasing the contribution of RAG-retrieved embeddings (e.g., 1:2 or 1:5) led to declines in AUC (0.8943 and 0.8931, respectively), although these ratios showed competitive specificity (0.8662 and 0.8779) and MCC (0.5273 and 0.5739). Similarly, emphasizing the original embeddings (e.g., 2:1 or 5:1) also resulted in lower performance, with the 5:1 ratio showing the lowest AUC (0.8596) and MCC (0.4629), alongside a drop in sensitivity (0.7671). These results indicate that an equal balance of original and retrieved embeddings (1:1) provides the most effective synergy, while overemphasizing either source compromises the model’s ability to leverage complementary information.

### Comparison of Performance with Different Classifiers

In Table 8, we have compared the performance of the multiwindow CNN model with traditional classification models, including KNN, RF, and SVM, all of which utilize a RAG-based similarity retrieval strategy. Both mCNN and RF demonstrated strong discriminative power, with mCNN achieving an AUC of 0.9074 and RF achieving a comparable AUC of 0.9038. However, the balanced accuracy (BACC) and MCC metrics reveal notable differences between the two models. Specifically, mCNN achieved a BACC of 0.8587 and an MCC of 0.6407, compared to RF’s 0.6646 and 0.4739, respectively. Furthermore, mCNN’s sensitivity (0.7808) was markedly higher than RF’s (0.3425), highlighting its superior ability to identify true positives without compromising specificity (0.9365). While RF remains a competitive classification model with strong AUC performance, mCNN provides a more balanced and robust classification across various metrics, making it particularly well-suited for this task.

**Table 8:** Comparison of Performance with Different Classifiers

classifier	BACC	sensitivity	specificity	accuracy	MCC	AUC
**mCNN**	**0.8587**	**0.7808**	**0.9365**	**0.9195**	**0.6407**	**0.9074**
RF	0.6646	0.3425	0.9866	0.9165	0.4739	0.9038
SVM	0.4620	0.7534	0.1706	0.2340	–0.0618	0.2128
KNN	0.5891	0.1781	1.0	0.9106	0.4023	0.8407

These comparisons underscore that the incorporation of the RAG-based similarity retrieval strategy with the multiwindow CNN model appears to be a crucial factor in enhancing the model’s predictive power. This makes it a more reliable tool than traditional classifiers for this specific application. A ROC curve for the performance of predicting IL-6-inducing peptides with different classifiers is presented in Figure 4b.

### Comparison of Performance with Previous Works

As shown in Table 9. In order to compare the performance of different existing methods, including MVIL6, StackIL6 and IL6Pred. All the performance comparisons with previous work were adopted from the same benchmark data set from the IL6Pred study. In the independent test results, the proposed multiwindow CNN model achieves the best performance of MCC 0.641 and AUC 0f 0.907. In comparison with MVIL6, the most recent method for identifying IL-6 inducing peptides, our method shows significant improvements in terms of MCC and AUC, with an increase of 17.6% in MCC and 2.4% in AUC. Additionally, our method demonstrates the highest specificity (0.937) and accuracy (0.920), indicating superior discriminative power and predictive accuracy. While MVIL6 shows competitive sensitivity (0.863), it lags behind in MCC and AUC, highlighting the robustness of our approach in identifying IL-6-inducing peptides.

**Table 9:** Comparison of Performance with Previous Works^a^

method	BACC	sensitivity	specificity	accuracy	MCC	AUC
**our method**	**0.859**	**0.781**	**0.937**	**0.920**	**0.641**	**0.907**
MVIL6	0.834	0.863	0.804	0.834	0.465	0.883
StackIL6	0.795	0.849	0.741	0.795	0.393	0.841
IL6Pred	0.743	0.753	0.732	0.743	X	0.830

a The results for comparison with previous methods are taken from Table 1 of MVIL6 paper.^12^

## Conclusion

In this study, we introduced RAG_MCNNIL6, an innovative deep learning framework that integrates Retrieval-augmented generation (RAG) with multiwindow convolutional neural networks (MCNNs) for the accurate prediction of IL-6 inducing epitopes. Through the strategic combination of PLM embeddings and a RAG-based similarity retrieval approach, our model effectively captures both global and local sequence patterns, significantly enhancing its predictive power compared to traditional methods.

Our method uses embeddings from protein language models (PLMs) combined with a RAG-based similarity retrieval approach. With the use of a curated database containing a wide range of interleukin-inducing peptides, by combining the embedding of the original query sequence with the averaged embeddings derived from the top similar sequences retrieved from the database. The new fusion embeddings.

We conducted a comprehensive evaluation of RAG_MCNNIL6 on the benchmark data sets and found that it outperformed existing methods in key performance metrics, such as sensitivity, specificity, accuracy, Matthews correlation coefficient (MCC), and area under the ROC curve (AUC). In independent testing, the model achieved an MCC of 0.641 and an AUC of 0.907, representing a significant improvement over prior models such as MVIL6 and StackIL6.

These findings underscore the effectiveness of incorporating contextual information through the RAG framework and highlight the potential of multiwindow scanning in deep learning architectures for epitope prediction. The results suggest that RAG_MCNNIL6 offers a robust and reliable tool for advancing research and therapeutic development in IL-6-mediated diseases, potentially aiding in the design of more effective epitope-based vaccines and immunotherapies.

While this study focused on the prediction of IL-6 inducing epitopes, the underlying principles of RAG_MCNNIL6 offer a promising framework for broader application. Future research will explore the adaptability of this model to predict epitopes for other cytokines involved in inflammatory responses and immune regulation, potentially uncovering shared sequence features or unique predictive patterns. Furthermore, investigations into alternative RAG architectures, such as incorporating dense retrieval methods or knowledge graph integration, could further enhance the retrieval of relevant contextual information.

The precise identification of IL-6 inducing epitopes through RAG_MCNNIL6 has significant implications for vaccine design, enabling the development of more targeted immunogens that elicit a stronger and more focused immune response. While our model provides a significant tool for identifying IL-6 inducing epitopes, it is important to acknowledge the need for further investigation into the extent of their induction potential. Certain strongly inducing epitopes may pose a risk for the development of cytokine storms. Future research should focus not only on epitope identification but also on evaluating the degree of IL-6 induction, perhaps by employing computational methods to assess epitope binding affinity and downstream pathway activation, allowing for selection of the safest and most efficacious sequences. Furthermore, strategies to modulate the immune response, such as using adjuvants that do not promote excessive IL-6 secretion, or to incorporate specific epitopes from IL-6 suppressive regulatory proteins, are important considerations to mitigate the potential for cytokine storm. Ultimately, in vitro and in vivo experiments will be necessary to validate the safety and efficacy of epitope-based vaccines designed based on these predictions.

This work was partially supported by the National Science and Technology Council, Taiwan under grant no. MOST 110-2221-E-155-038-MY2 and NSTC 112-2221-E-155-020-MY3.

## Data Availability

The authors confirm that the data supporting the findings of this study are available within the article. Data are available at https://github.com/B1607/RAG_MCNNIL6.

## Notes

The code and data for this work are available at https://github.com/B1607/RAG_MCNNIL6.
